# Prevalence and factors associated with antenatal care utilization in Ethiopia: an evidence from demographic health survey 2016

**DOI:** 10.1186/s12884-020-03236-9

**Published:** 2020-09-11

**Authors:** Berhan Tsegaye, Mohammed Ayalew

**Affiliations:** 1 Department of Midwifery, College of Medicine and Health Science College, Hawassa University, Hawassa, Ethiopia; 2 Department of Nursing, College of Medicine and Health Science College, Hawassa University, Hawassa, Ethiopia

## Abstract

**Background:**

Ethiopia is one of the sub-Saharan African country with high maternal mortality ratio (MMR). According to Ethiopian demographic health survey (EDHS) 2016 report, MMR is 420 among 100,000 live births. Antenatal care utilization is a key intervention to reduce these deaths through problem detection and treatment, promotion of health seeking behavior, and preparing pregnant women for birth. Therefore, this study aimed to assess prevalence and factors associated with antenatal care service utilization in Ethiopia in 2016.

**Methods:**

Secondary data analysis was done on EDHS 2016. It was a stratified, two-stage, and cluster sampling design. Analysis has been restricted to antenatal care utilization among women who delivered at least one time in the past five years. Data were weighted to correct sampling bias. Moreover, complex data analysis was done. Bi-variate and multivariable logistic regression analyses were carried out. Adjusted odds ratio with 95% confidence interval was computed and P-value less than 0.05 considered as a statistically significance level for identification of association.

**Results:**

Prevalence of antenatal care utilization was 62.8% [95%CI: 60.9, 64.6] in this study. Maternal educational status of primary school (AOR = 1.8,95%CI:1.2, 2.6), maternal educational status of secondary school (AOR = 4.4,95%CI: 1.1, 17.3), women who listen radio less than 1 per week (AOR = 1.9,95%CI:1.12,3.34), women who listen radio at least 1 per week (AOR = 2.6,95%CI:1.4,4.8), women in rich wealth quintile (AOR = 1.9,95%CI: 1.1, 3.2) were factors positively associated with antenatal care utilization. However, women who had traditional belief (AOR = 0.1,95%CI:0.02,0.49), and women who had five children and above (AOR = 0.6,95%CI: 0.3, 0.9) were factors associated negatively with antenatal care utilization.

**Conclusions:**

Prevalence of antenatal care utilization is still low in Ethiopia in 2016. Maternal higher maternal educational status, frequent radio listening, higher wealth quintile, traditional belief, and greater number of children were found to be associated significantly with antenatal care utilization. Consequently, socio-economic status should be enhanced, information should be accessed by women about antenatal care utilization and family planning service through mass media. Furthermore, intensive community education program should be designed for traditional believers to increase uptake of antenatal care by stakeholders.

## Background

Globally, maternal mortality and morbidity encompasses a greatest challenge to human development. Almost all of maternal deaths are occurred in low and middle income countries [1]. Maternal mortality rate was declined by 45% from 380 to 210 deaths per 100,000 live births between1990 and 2013 in the world [2]. First, the finished millennium development goal agenda 5 targeted to reduce maternal deaths by 75% between 2000 and 2015 [3]. Next, sustainable Development Goal 3.1 sets a specific target of MMR reduction below 70 by 2030 in the world [4]. Most of these maternal deaths occur due to causes directly related to pregnancy [5]. Fortunately, these maternal deaths can be prevented through provision of antenatal care, and institutional based delivery service [6].

Antenatal care often presents the first contact as an opportunity for women link with formal health services. It is an entry point for integrated care, safe home practices, improved health seeking behaviors, and a strong bond of women with complications to higher institution [7]. Although world health organization recommended four antenatal visits for low risk pregnancy, the optimal number of antenatal care visits are still debatable [8]. It is depends not only on effectiveness but also on feasibility and other barriers to antenatal care access and supply in low-income countries [9]. Most of pregnant women start antenatal care visit late in pregnancy due to many reason in developing countries [10]. The new focused antenatal care approach emphasizes on the quality of care rather than quantity [11]. Moreover, demographic health survey program use three different types of indicators to compare antenatal care service utilization across different countries. Fore example, ANC visit only one time in the last childbirth is one of the most important indicator [12]. The government of Ethiopia adopted several cost effective interventions to increase antenatal care utilization such as: health extension program was excuted by the government, skilled health providers were extensively trained, antenatal services became free from charge, and health infrastructures were expanded [10]. However, antenatal care utilization still a major challenge in Ethiopia. According to Ethiopian demographic health survey report, the coverage of antenatal care was 34% ranging from urban (76%) to rural (26%) women in 2011 [8].

Antenatal care service utilization was significantly influenced by maternal age and husband attitude towards antenatal care utilization [13, 14]. In addition to this, women missed opportunities for care due to client socio-demographic and reproductive health factors. Mothers and children may face risks because of limited or close to term antenatal care visits [15]. It is invaluable to understand the various factors which affect the utilization of antenatal care so that the respective programs are implemented more effectively and efficiently. If we fail to identify these bottlenecks, we can not improve antenatal care utilization. Although studies have been conducted previously about antenatal care utilization, they focused on antenatal utilization particularly on women who complete four antenatal visits.

Therefore, this study aimed to assess prevalence and factors associated with antenatal care utilization at least once in the past five years before Ethiopian demographic health survey of 2016.

## Methods

Ethiopia is divided into 9 regional states, and two city administrations. These regional states and administrations are further sub-divided into 75 zones, 551 districts(woredas) and 10,000 Kebeles. In 2004/05, there were 126 hospitals, 519 health centers, 1,797 health stations, 2899 health posts and 1,299 private clinics. There are limited number of health institutions and medical supplies in the countr. Furthermore, resource disparity between urban and rural have made difficult to improve access to health care services in Ethiopia [16]. Primary health care service comprises the following care package: preventive, promotive and curative services. In Ethiopia, health sector development plan-I have introduced having a four-tier health system for health service delivery. It consists of different level of health institutions from lowest to highest: one health center and five satellite health posts, distric hospital, zonal hospital, and specialized hospitals. Antenatal care services are primarily offered in health centers free of charge for pregnant women. Health extension workers refer women with danger signs to higher health facilities [16].

Dependent variable was antenatal care service utilization. It is a binary outcome variable. Those women who did not utilize antenatal care service was assigned as ‘0’ ,but those who utilize antenatal care utilization was assigned as ‘1’ during analysis. In this study, antenatal care utilization was defiened as: Women who visited health facility at least once during their last pregnancy were considered as antenatal care service utilizer, otherwise,not.Thus,study participants were asked whether they utilized antenatal care or not during pregnancy five years before data collection time. Independent variables are categorized into two main groups. These includes socio-economic and reproductive health variables. Socio-demographic characteristics include study participant’s age, place of residence, educational status, husband educational status, religion, ethnicity, marital status and wealth index. Moreover, reproductive health characteristics consisted of the following variables. Age at first pregnancy, number of alive children, current pregnancy status, place of delivery and frequency of listening radio. We have utilized principal component analysis to construct wealth index. Diffent type of house hold assets are variables used in principal component analysis. Thus include size of production land, livestock and house building material. Hence,wealth quantile is divided into five levels: Poorest, poorer, medium, richer and richest [17]. Wealth index was created in three consuctive steps.First,the subset of urban and rural households indicators were built separetly. Second, scores were created separately for each household both in urban and rural. Finally, a national wide wealth index was created by combination of both urban and rural [18].

**Fig. 1:**
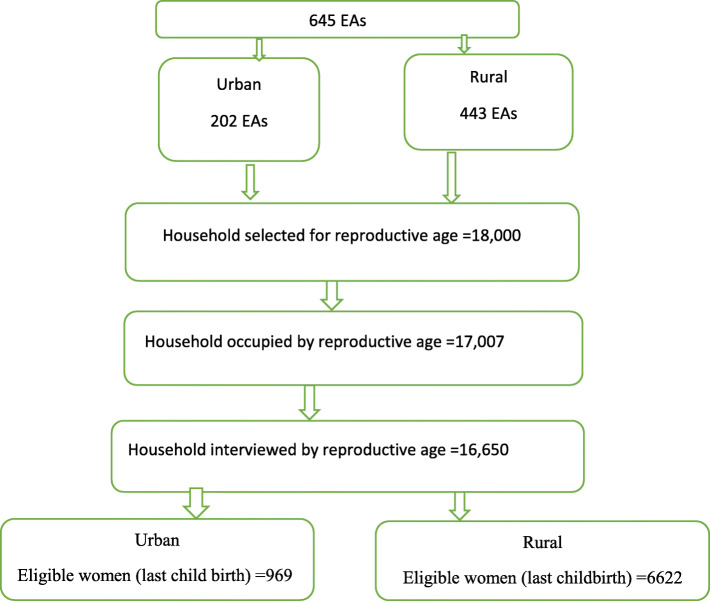
Schematic presentation of sampling strategy of antenatal care utilization among women with last childbirth in Ethiopia (EDHS 2016)

The source of sampling frame for EDHS 2016 was Ethiopian population, and housing census of 2007. The smallest administrative unit (Kebele) was sub-divided into census enumeration areas (EAs) during housing census in 2007. Hence, sampling frame contained complete lists of enumeration areas. It contained a number of important information: For instance, location, type of residence, and estimated number of residential households. Therefore, EAs were primary sampling units of EDHS 2016. Each EA consisted of 181 households. Stratified, two stage cluster sampling strategy was applied in EDHS 2016. First, 645 EAs were selected, and allocated proportionally based on the total number of enumeration areas throught the country both in rurban and rural areas. Consequently, 202 were selectd from urban area, and 443 were from rural areas. Second, a fixed number of 28 households per cluster/EA/ were selected using systematic random samping. As a result, a total of 18,008 households were selected. From these, 17,067 households were occupied by women of reproductive group. Effective interview was conducted over women at 16,650 househlds. However, only 16,583 eligible reproductive age women were existed in the selected househods. Furthermore, only 15,683 study participants gave full response making a response rate of 95% [19]. In the current analysis, women who had at least one child birth within the past five years before data collection were selected. Due to this, a total of 7591 participats were selected, and analyzed for antental care utilization. Figure 1 indicates the extraction of samples from EDHS for this study (Insert Fig. 1).

Data were analyzed using SPSS version 22. For study participants who gave birth more than one time within five years, the most recent birth was considerd for analysis. Furthermore, we restrict the analysis to the most recent birth to minimize the recalling bias. Then, important variables were selected, re-categorized and coded again to make them comparable across studies. Ideally, both bias and variance should be kept minimum in complex survey. Consequently, we applied sampling weight to minimize bias to compensate for unequal probability of selection among geographic strata.Weight variarable was created by dividing the individual woman variable (V005) by 1,000,000. Hence, prevalence of antenatal care utilization was calculated after weighing EDHS data. Stratification and clustering were used to compute standard error. Complex data analysis is not included in SPSS package. Therefore, SPSS will assume that our data come from simple random sampling. Hence, standard error will be underestimated. For this reason, dataset was adjusted for complex analysis. In other words, we told SPSS to account of sample design, and calculating standard error. We have done it in the following steps: First, we have computed weight to measure point estimates by dividing individual women (v005) by 1,000,000. Second, we have created plan file by using three variables needed to set up complex samples. These include the following variables in the dataset: primary sample unit (021), sample strata (v022), and weight created in step 1. Third, we have fixed sampling with replacement as estimator assumption. Finally, we have analyzed the plan file created in these three steps to identify factors associated with antenatal care utilization.

Chi-square test was performed to observe any association betewin independent variables and an outcome variable. First, we performed binary logistic regression analysis to identify variables associated with antenatal care service utilization. In binary logistic regression analysis, we took variables with p-value less than or equal to 0.05 method into multivariable logistic regression analysis to control cofounders. Then, varaibles which had significant association with antenatal care utilization were identified based on Adjusted Odds Ratio (AOR) and p-value less than 0.05 in the multivariable logistic regression analysis model. We used enter method to identify associated factors in crude odds ratio and manual backward stepwise method to compute AOR in multivariable logistic regression model. Descriptive statistics was conducted by the use of texts and tables.

## Results

We selected a total of 7591 study participants from women data file of EDHS 2016. Then, we analyzed prevalence and factors associated with antenatal care utilization. Particularly, this analysis was restricted to women who delivered at least once before EDHS 2016. All the selected study participants have given full response making a response rate of 100%. Table 1 showed the socio-demographic charactestics of the study participants. The mean age of the study participants was 28 ± 9 years. Majority 7116 (92.4%) of them were in the age range of 25–39 years. Nearly half 3369 (forty-four percent) of them were not educated. From the total study participants, more than half 4075 (53.7%) of them were jobless. Majority of the respondents 6622 (82.9%) were rural residents. Furthermore, most of the study participants 7109 (93.7%) were married (Insert Table 1).

**Table 1:** Demographic and socio-economic charactestics of study population. (*N* = 7591)

	Non utilization of ANC		Utilization of ANC		Chi-square p-value ^a^
**Age**	Weighted (N)	Weighted (%)	Weighted (N)	Weighted (%)	**0.003**
15–24	2638	37.6	4377	62.4	
25–49	180	31.3	395	68.7	
**Religion**					**0.000**
Orthodox	852	29.6	2030	70.4	
Catholic	30	41.7	42	58.3	
Protestant	602	36.3	1049	63.5	
Muslim	1251	44.3	1573	55.7	
Traditional	45	46.4	51	53.6	
Other	39	60	26	40	
**Marital status**					0.333
Married	2628	37	4481	63	
Single	64	41.8	89	58.2	
Divorced	42	43.8	54	56.2	
Widowed	85	36.5	148	63.5	
**Ethnicity**					**0.000**
Amhara	573	30.2	1327	69.8	
Oromo	1415	48.0	1533	52.0	
Somali	140	54.9	115	45.1	
Sidama	74	26.0	211	74.0	
Others^*^	617	28.0	1586	72.0	
**Educational status**					**0.002**
No education	1227	36.4	2142	63.6	
Primary	930	37.3	1561	62.7	
Secondary	422	39.4	650	60.6	
Higher	240	36.5	418	63.5	
**Occupation**					**0.355**
No work	1564	38.38	2511	61.62	
Government worker	673	37.51	1121	62.49	
Farmer	428	32.33	896	67.67	
Private	94	36.43	164	63.57	
Daily worker	59	42.75	79	57.25	
**Wealth index**					**0.000**
Poorest	857	51.91	794	48.09	
Poorer	719	43.47	935	56.53	
Middle	592	37.28	996	62.72	
Rich	460	32.24	967	67.76	
Richest	191	15.04	1079	84.96	
**Marital status**					0.333
Married	2628	36.97	4481	63.03	
Single	64	41.83	89	58.17	
Divorced	42	43.75	54	56.25	
Widowed	85	36.48	148	63.52	
**Total family size**					**0.000**
1–5	1123	30.88	2514	69.12	
6 and above	2819	37.14	4772	62.86	
**Residence**					**0.000**
Urban	94	9.70	875	90.30	
Rural	2819	37.14	4771	62.86	

^a^statistically significant *p*-value are in bold

Regarding about reproductive charactersticies, most of the study participants (62.3%) were married at the age of 15–24 years. Furthermore, more than a quarter 1965 (25.9%) of the study participants had five and above children. In addition, more than half (62.9%) of the study participants had antenatal follow up in their last childbirth. Nearly all 6820 (90.8%) of the study participants were not pregnant at the time of data collection. Majorities (72.3%) of the study participants did not listen radio totally. Moreover, majority 7016 (92.4%) of the study participants were also found in the age range of 15–24 years. More than half (67.3%) the study participants were aso delivered at health institution (Insert Table 2).

**Table 2:** Reproductive health charactestics of study population. (*N* = 7591)

	Non utilization of ANC		Utilization of ANC		X^2^ p-value ^a^
**Age at first birth**	Weighted (N)	Weighted (%)	Weighted (N)	Weighted (%)	**0.003**
15–24 years	2446	85.38	419	14.62	
25–49 years	180	31.36	394	68.64	
**Place of birth**					
Home	2555	50.04	2551	49.96	**0.000**
Health institution	263	10.42	2260	89.58	
**Number of children**					**0.000**
1–2 children	660	25.12	1967	74.88	
3–4 children	752	37.66	1245	62.34	
5 and above	1406	47.420	1559	52.58	
**Current pregnancy**					
No	2439	35.76	4381	4381	**0.000**
Yes	379	49.28	390	390	
**Frequency of listening radio**					**0.000**
Not at all	2329	42.41	3162	57.59	
Less than once a week	237	23.01	793	76.99	
At least once a week	252	23.57	817	76.43	

^a^statistically significant *p*-value are in bold

Regarding about factors associated with antenatal care utilization, twelve factors were statistically associated with antenatal care utilization in binary logistic regression.These include the following variables:Age, religion,ethinicity,educational status,wealth index ,total family size and residence, frequency of listening radio, place of delivery,current pregnancy,age at first birth and number of children alive. But, as Table 3 showed that only five variables were significantly associated with antenatal care utilization in multivariable logistic regression model. These were: Educational status, wealth index, number of children alive, frequency of listening radio and religion. Educational status was one of the significantly associated factors. Study participants whose educational status of primary school were 1.8 times more likely to utilize antenatal care service than non-educated (AOR = 1.8,95%CI, 1.2, 2.6, *p* < 0.001). Moreover, those participants whose educational status of secondary education school were 4.4 times more likely to utilize antenatal care service than non-educated (AOR = 4.4,95%CI, 1.1, 17.3,*p* < 0.001). Study participants who listened radio less than one week were 1.9 times more likely to utilize antenatal care than those who did not listen at all (AOR = 1.9,95%CI, 1.12, 3.34,*p* < 0.002). Besides, those who listen radio at least one per week were 2.6 times more likely than who did not listen radio totally (AOR = 2.6,95%CI, 1.4, 4.8,*p* < 0.001). Compared to study participants in the poorest wealth quantile, study participants in rich wealth quintile had 1.9 fold higher odds of antenatal care utilization (AOR = 1.9,95%CI,1.1, 3.2, *p* < 0.001). The likelihood of antenatal care utilization was decreased by 90% among traditional believers than orthodox Christianity followers (AOR = 0.1,95%CI, 0.02, 0.49, *p* < 0.001). This analysis revealed that the chance of antenatal care among study participants who had five and above children were by 40% less than study participants with one to two children (AOR = 0.6,95%CI, 0.3, 0.9, *p* < 0.003) (Insert Table 3).

**Table 3:** Factors associated with antenatal care service utilization among women who gave birth five years before Ethiopian demographic health survey 2016 (*N*=7591)

		Antenatal care utilization		COR [95%CI]	AOR [95%CI]	*p*-value
Variables		No	Yes			
Respondents education	No education	2211	2580	1	1	
	Primary	573	1577	2.3 (2.1, 2.6)*	1.8 (1.2, 2.6)**	0.001
	Secondary	32	388	10 (7.2, 14.0)*	4.4 (1.1, 17.3)**	0.001
	Higher	30	200	5.7 (0.8, 6.8)	4.7 (0.3, 7.8)	
Frequency of listening radio	Not at all	2329	3162	1	1	
	Less than 1/week	237	793	2.4 (2.1, 2.8)*	1.9 (1.12, 3.34)**	0.002
	At least 1/week	252	817	2.3 (2.0, 2.7)*	2.6 (1.4, 4.8)**	0.001
Wealth index	Poorest	857	794	1	1	
	Poorer	719	935	1.4 (1.2, 1.6)*	1.2 (0.7, 1.7)	
	Middle	592	996	1.8 (1.5, 2.0)*	1.0 (0.6, 1.7)	
	Rich	460	967	2.2 (1.9,2.9)*	1.9 (1.1, 3.2)**	0.001
	Richest	191	1079	6.1 (5.0,7.3)*	0.9 (0.3, 2.2)	
Religion	Orthodox	852	2030	1	1	
	Protestant	632	1091	0.72 (0.63,0.82)*	0.9 (0.6,1.5)	
	Muslim	1271	1573	0.52 (0.47,0.58)*	0.7 (0.5,1.1)	
	Traditional belief	83	77	0.39 (0.28,0.53)*	0.1 (0.02,0.49)**	0.001
Total number of alive children	1–2	766	2104	1	1	
	3–4	814	1330	0.59 (0.52,0.62)*	1.1 (0.7, 1.2)	
	5 and above	1220	1307	0.39 (0.34,0.43)*	0.6 (0.3, 0.9)**	0.003

Key - *= statistically significant at binary logistic regression ,1 = References group, *CI* confidence Interval, *COR* rude Odds Ratio, *AOR* Adjusted Odds Ratio, *ANC* Ante-Natal Care

## Discussion

Prevalence of antenatal care utilization was 62.8% (95%CI,60.9–64.6) in this study. This finding is consistent with finding of the study done in North Maharashtra (64.2%) [20, 21]. The possible rational for this consistency might be due to the following reasons: Study participants were in similar condition with socio-demographic status, awareness level about antenatal care service,and common traditional practice promoting home treatment. However, this finding is higher than finding of EDHS 2011 (34%) [22]. The possible explanation for this inconsistency might be due to two main reasons: First, the main factor which increased antenatal care service utilization in the this study might be that analysis was done on any women who received antenatal care follow up by any health professional. For,example, most of the antenatal care utilization were given by health extension workers in rural Ethiopia. However, findings of other studies have shown about antenatal care utilization given by skilled delivery attendant only. Around 80% of Ethiopian women live in rural area in which most of women could not get access of skilled antenatal care service. Ethiopia trained health extension workers to make access to maternal health care including antenatal care, clean delivery service and post-natal care. These professionals are not listed under skilled providers. Second, socio-economic status of participants was improved in this study than participants of studies conducted before five years. In conclusion, infrastructure were more accessible, women were more awared about the service, and residence places were more urbanized in the current study than before .This finding is also higher than findings of studies conducted in other countries: Rwanda (54%) [23] ,LAO PDR (53.9%) [24], and Bangladesh (32.6%) [25]. This might be due to more awareness, better access of service, and better understanding of service utilization by participants in this study. Besides, antenatal care utilization was restricted to the most recent pregnancy to recall easily. To conclude, all of these factors increased antenatal care utilization in the current study. Education status, frequency of listening of radio, wealth quintile, religion, and number of children were factors statistically associated with antenatal care utilization. Study participants who attended primary and secondary school education were 1.8 and 4.4 times more likely to utilize antenatal care service respectively as compared to women who had no formal education. This finding is in line with reports of other previous studies [26–30]. The possible justification of this finding might be that mothers who are educated more, tend to use antenatal care utilization, have better understanding of information and have better knowledge about importance of the service [29]. Moreover, educated women are more likely to improve independence, self-confidence and ability to make decisions about their own health. It is also likely that literate women seek out higher quality services and greater ability to use health care inputs that offer better care [31]. According to this finding, antenatal care service was more utilized by women of rich wealth index than women in poorest wealth quintile. This finding is supported by findings of previous studies [26, 29, 32]. The possible rational for this similarities might be that antenatal care utilization need both direct and indirect cost. Although antenatal care service is provided for all pregnant women free of charge to avoid these financial barriers [30], this could not completely avoid the financial constraints looking for health care services during a pregnancy. Because, pregnant women were charged for transportation while traveling to reach distant health institutions. This could hinder the women for an early initiation and subsequent antenatal care visits. When study participants travel to the health institution through a long distance, they pay a lot of money for transportation for relatives accompying them. Therefeore, indirect costs might be the possible barrier even though the services are provided freely. Women of higher parity, who have more than five children, were 40% less likely to use antenatal care service than their counter parts. Similar findings were also reported by other previous studies [1, 33]. The possible reason might be due to several factors: traditional practice is common among women who had many children, women of high parity are economical less disadvantaged group, and relative lesser complications are occurred among women of high parity due to their profound birth expriance. So, they fail to use antenatal service than their counterparts [1]. In short, high parity women are less motivated to go to health institutions for antenatal care utlization [2, 5]. Regarding about habit of listening radio, study participants who listened radio frequently utilized antenatal care service more than their counterparts. This finding is consistent with another finding [34, 35]. The possible explanation for this consistency might be due to the fact that accessing information about the benefit of antenatal care utilization and danger sign of pregnancy could assist women in making decision for utilization of service. Traditional believers were less likely to utilize antenatal care service than orthodox Christianity followers. This finding is consistent from studies conducted in Ngeria [36]. The possible explanation might be that traditional belief leaders might preclude modern health serice utilization for their followers due to their longstanding perception,custom and tradition. Besides,they may also encourage them to conduct certain practices related with the faith to have good pregnancy outcome.

This study has a number of limitations. First, the analysis is limited to women who had antenatal care utilization of only one visit or not. Second, this study was community based so that it did not address health institution factors of antenatal care utilization. Finally, the cross sectional nature of the study could not assist the temporal relationship of variables. Therefore, further study should be studied to identify factors related to health institution factors. In addition, antenatal care utilization should be conducted based on WHO definition at national level. Moreover, longitudinal studies should be studied which address comprehensive variables.

## Conclusions

Antenatal care utilization is slightly higher than prevous national findings in Ethiopia in this study. However, it is not still satisfactory. Women with higher education status, more frequent listening of radio, and being rich in wealth quintile were factors positively influence antenatal care utilization. On the other hand, follower of traditional belief, and having five or more children were factors negatively associated with antenatal care service utilization. Due to this reason, ministry of health of Ethiopia, policy makers, and program designers should create awareness about antenatal care utilization through different media. Furthermore, improving socio-economic status, designing, and implementing different family planning programs should be mandatory. In addition, intensive community education program should be designed for traditional belief followers to increase uptake of antenatal care service. In summary, awareness creation and improving living condition of women could increase uptake of antenatal care utilization. Consequently, maternal mortality and morbidity can be reduced and target of SDG can be meet in Ethiopia.

## Data Availability

Permission to access database was officially obtained. The database was available at a official website of DHS which is at https://dhsprogram.com.

## Abbreviations

CI: Confidence Interval
DHS: Demographic and Health Survey
COR: Crude Odds Ratio
EA: Enumeration Area
ANC: Ante-Natal Care
CSA: Central Statistical Agency

## References

[1] Organization WH (2005). The World health report: 2005: make every mother and child count.

[2] Lawson GW, Keirse MJ (2013). Reflections on the maternal mortality millennium goal. Birth.

[3] Afework, M.F., Achieving the maternal health millennium development goals in ethiopia: where are we and what needs to be done? 2010, soc latinoamer especialistas mamiferos acuaticos c/o salvatore siciliano ….

[4] Yamin AE, Boulanger VM (2013). Embedding sexual and reproductive health and rights in a transformational development framework: lessons learned from the MDG targets and indicators. Reproductive Health Matters.

[5] Kvale G, Hinderkar OB, Ulstein SG, Bergsjo M. P, Maternal deaths in developing countries: a preventable tragedy. Norsk Epidemiol. 2005;15:141–9.

[6] McCaw-Binns A, Grenade JL, Ashley D (1995). Under-users of antenatal care: a comparison of non-attenders and late attenders for antenatal care, with early attenders. Soc Sci Med.

[7] Kassebaum NJ (2016). Global, regional, and national levels of maternal mortality, 1990–2015: a systematic analysis for the Global Burden of Disease Study 2015. The Lancet.

[8] Carroli G (2001). WHO systematic review of randomised controlled trials of routine antenatal care. The Lancet.

[9] Lincetto O, Gomez M-A, Munjanja P. S. Chapter 2: Antenatal care. In: Lawn J, Kerber K, editors, Opportunities for Africa’s newborns: practical data, policy and programmatic support for newborn care in Africa. Geneva: World Health Organization. 2006(51–62.).

[10] Van Eijk AM (2008). Reproductive health issues in rural Western Kenya. Reproductive health.

[11] Organization WH (2004). Antenatal care in developing countries: Promises, achievements and missed opportunities.

[12] B P, Health Service Resources Rural, determinants of infant death in Study, Bangladesh: An empirical. Soc Sci Med, 1991. 32: p. 43–49. 10.1016/0277-9536(91)90125-v 2008620

[13] Woldie M. orignal article factors influencing antenatal care service utilization in hadiya zone. 10.4314/ejhs.v20i2.69432 PMC3275839 22434964

[14] Mesganaw F, Shamebo OG. D., Determinants of ANC Attendance and Preference of Site or Delivery in Addis Ababa. Ethiopia Journal of Health Development. 1990;6:17–21.

[15] F Jane (2005). Feasibility of introducing a comprehensive integrated package of antenatal care services in rural public clinics in South Africa.

[16] Oladapo OT, Osiberu MO (2009). Do sociodemographic characteristics of pregnant women determine their perception of antenatal care quality?. Maternal Child Health J.

[17] Rutstein SO (2008). The DHS Wealth Index: Approaches for rural and urban areas.

[18] CSA-Ethiopia I, International (2012). Ethiopia Demographic and Health Survey 2011.

[19] Agency CS, The DHS Program ICF Rockville, Maryland, USA. 2017.

[20] Mumbare SS, Rege R (2011). Ante natal care services utilization, delivery practices and factors affecting them in tribal area of North Maharashtra. Indian journal of community medicine: official publication of Indian Association of Preventive Social Medicine.

[21] Kushwaha P (2016). Utilization of antenatal care services in periurban area of Aligarh. International Journal of Medical Science Public Health.

[22] Tarekegn SM, Lieberman LS, Giedraitis V (2014). Determinants of maternal health service utilization in Ethiopia: analysis of the 2011 Ethiopian Demographic and Health Survey. BMC Pregnancy Childbirth.

[23] Rurangirwa AA (2017). Determinants of poor utilization of antenatal care services among recently delivered women in Rwanda; a population based study. BMC Pregnancy Childbirth.

[24] Ye Y (2010). Factors affecting the utilization of antenatal care services among women in Kham district, Xiengkhouang province, Lao PDR. Nagoya J Med Sci.

[25] Ghose B (2017). Women’s decision-making autonomy and utilisation of maternal healthcare services: results from the Bangladesh Demographic and Health Survey. BMJ open.

[26] Nwosu EO, Author C, Urama NE (2012). Determinants of Antenatal Care Services Utilisation in Nigeria. Developing Country Studies.

[27] Regassa N. Antenatal and postnatal care service utilization in southern Ethiopia: a population-based study. African Health Sciences Vol, 2011. 11. PMC3260999 22275929

[28] Amentie M, Abera M, Abdulahi M. Utilization of Antenatal Care Services and Influencing Factors among Women of Child Bearing Age in Assosa District, Benishangul Gumuz Regional State, West Ethiopia. Global Journal of Medical Research: E Gynecology and Obstetrics, 2015. 15.

[29] Paudel DP, Nilgar BR, Bhandankar M (2013). Antenatal care service utilization and contributing factors: a community based study in rural Belgaum, Karnataka, India. IOSR Journal of Dental Medical Sciences.

[30] Abegaz KH. Exploring trend and barriers of antenatal care utilization using data mining: evidence from EDHS of 2000 to 2016. BioRxiv, 2018: p. 351858.

[31] Mekonnen Y, Asnaketch AMMY. M., Utilization of Maternal Health Care Services in Ethiopia. Maryland: ORC Macro: Calverton; 2002.

[32] Kushwaha P, et al., Utilization of antenatal care services in periurban area of Aligarh. International Journal of Medical Science and Public Health, 2016. 5.

[33] Kvåle G, et al., Maternal deaths in developing countries: A preventable tragedy. Norsk Epidemiologi, 2005. 15(2).

[34] Acharya D (2015). Impact of mass media on the utilization of antenatal care services among women of rural community in Nepal. BMC Res Notes.

[35] Birmeta K, Dibaba Y, Woldeyohannes D (2013). Determinants of maternal health care utilization in Holeta town, central Ethiopia. BMC Health Serv Res.

[36] Doku D, Neupane S, Doku PN (2012). Factors associated with reproductive health care utilization among Ghanaian women. BMC international health human rights.

